# Massive Transfusion Protocol and Outcome of Patients with Acute Variceal Bleeding

**DOI:** 10.3390/jcm13092588

**Published:** 2024-04-28

**Authors:** Aryoung Kim, Dong Hyun Sinn, Byeong Geun Song, Myung Ji Goh, Sung Yeon Hwang, Ryoung-Eun Ko, Chi Ryang Chung, Chi-Min Park

**Affiliations:** 1 Department of Medicine, Samsung Medical Center, Sungkyunkwan University School of Medicine, Seoul 06351, Republic of Korea; 2 Department of Internal Medicine, Inje University Ilsan Paik Hospital, Goyang 10380, Republic of Korea; 3 Department of Emergency Medicine, Samsung Medical Center, Sungkyunkwan University School of Medicine, Seoul 06351, Republic of Korea; 4 Department of Critical Care Medicine, Samsung Medical Center, Sungkyunkwan University School of Medicine, Seoul 06351, Republic of Korea

**Keywords:** massive transfusion protocol, variceal bleeding, restrictive transfusion

## Abstract

**Background/Aims:** The massive transfusion protocol (MTP) can improve the outcomes of trauma patients with hemorrhagic shock and some patients with non-traumatic hemorrhagic shock. However, no information is available regarding whether MTP can improve the outcomes of acute variceal bleeding (AVB). This study aimed to determine the effects of MTP on the outcomes of patients with AVB. **Methods:** Consecutive patients (*n* = 218) with AVB who did not have current malignancy and visited the emergency room between July 2014 and June 2022 were analyzed. 42-day mortality and failure to control the bleeding were compared between patients with and without MTP activation. Additionally, propensity-score matching was conducted. **Results:** The amount of blood product transfused was higher in the MTP group. The 42-day mortality rate (42.1% vs. 1.5%, *p* < 0.001) and the rate of failure to control bleeding (36.8% vs. 0.5%, *p* < 0.001) were significantly higher in those who received blood transfusions by MTP. MTP was an independent factor associated with 42-day mortality in the multivariable-adjusted analysis (HR 21.05; 95% CI 3.07–144.21, *p* = 0.002, HR 24.04; 95% CI 3.41–169.31, *p* = 0.001). The MTP group showed consistently higher 42-day mortality and failure to control bleeding in all subgroup analyses, stratified by systolic blood pressure, hemoglobin level, and the model for end-stage liver disease score. The MTP group also showed higher 42-day mortality (42.9% vs. 0%, *p* = 0.001) and failure to control bleeding (42.9% vs. 0%, *p* = 0.001) in a propensity score-matched analysis (*n* = 52). **Conclusions:** MTP was associated with poor outcomes in patients with AVB. Further studies are needed to see whether MTP can be an option for patients with massive AVB.

## 1. Introduction

Acute variceal bleeding (AVB) is one of the fatal complications of liver cirrhosis [1,2]. Despite advances in management and therapy, AVB occurs in approximately half of the cirrhotic patients prior to transplantation, and mortality from each episode of AVB remains high, ranging from 5% to 25% [3,4,5]. The current approach to patients with AVB is a multimodal strategy aimed at controlling acute bleeding, preventing rebleeding, and lowering the 6-week mortality rate. For an AVB episode, endoscopic treatment within the first 12 h of admission [3], short-term administration of prophylactic antibiotics [6], and intravenous infusion of splanchnic vasoactive medications for 5 days is recommended [7]. In particular, limited red blood cell (RBC) transfusion is recommended by starting transfusion when hemoglobin is less than 7 g/dL and maintaining a goal of 7–9 g/dL [8,9].

Massive transfusion is defined as the rapid administration of a significant volume of blood components. The most prevalent definition of massive transfusion in adult patients is transfusion of more than 10 units of packed red blood cells (pRBC) within 24 h or 3 units of pRBC at any time within one hour after recognizing life-threatening hemorrhage [10,11]. The massive transfusion protocol (MTP) includes minimizing the use of crystalloid fluids and early administration of large amounts of blood components, particularly plasma and platelets, to mimic whole blood transfusion and improve coagulation profiles and death rates in trauma patients. It has been shown that the implementation of MTP can improve the outcomes of trauma patients [12,13,14]. There is growing interest in using MTP in non-trauma patients with massive bleeding, with inconsistent reports [15,16]. Acute blood loss and hemodynamic instability can occur in AVBs. However, there is no information on whether MTP can be used to improve the outcomes of patients with AVB. Given that the mortality rate for AVB remains high [3,4,5] and that we are in the era of guidelines emphasizing limited blood transfusion in patients with AVB as several studies have confirmed that limited blood transfusion improves mortality and morbidity when portal hypertension-related bleeding occurs [17,18], it is critical to determine whether administering MTP to patients with AVB would be helpful for improving their survival. In our institution, we have implemented MTP in the emergency room since July 2014, including in non-trauma patients. Herein, we report our experience with MTP in AVB patients.

## 2. Materials and Methods

### 2.1. Study Design, Setting, and Participants

**Figure 1:**
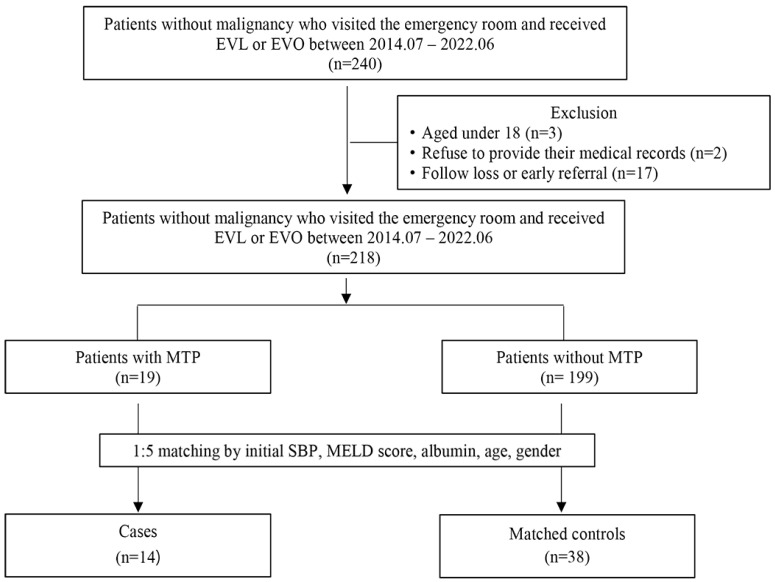
Study population diagram. *MTP, massive transfusion protocol; EVL, endoscopic variceal ligation; EVO, endoscopic variceal obliteration; SBP, systolic blood pressure; MELD, model for end stage liver disease*.

This was a single-center retrospective cohort study of AVB patients who did not have current malignancy and visited the Samsung Medical Center emergency room between 1 July 2014 and 30 June 2022. We investigated patients who do not currently have malignant tumors, given that malignant tumors are a large competing risk factor for mortality, and we aimed to investigate causes of death directly related to AVB. In our institution, MTP was implemented in July 2014. We screened 240 consecutive patients with AVB without current malignancy during the study period using electronic medical records. The time interval used to define an AVB episode was 5 days [2]. Varix included both gastric and esophageal varices and varix bleeding was defined based on the results of the endoscopic examination, if blood clots or white nipples appeared on the surface of varices, or if blood was found in the stomach without a potential bleeding focus other than esophageal or gastric varcies [17]. Among them, we excluded patients aged < 18 years (*n* = 3) and those who refused to provide their medical records for medical purposes (*n* = 2) since our hospital investigated their intent at the time of their visit. We further excluded 17 patients with early referral to another hospital (*n* = 7) because of their hometown and follow-up loss (not visited after discharge) (*n* = 10) since they were unable to assess the study’s result, which included the 42-day mortality rate and the rate of bleeding control failure. Finally, 218 adult patients with AVB without current malignancy were analyzed (Figure 1). This study was approved by the Institutional Review Board of Samsung Medical Center (approval no. 2022-12-058). The requirement to obtain informed consent was waived as we used only de-identified data routinely collected during hospital visits.

### 2.2. Variables and Measurements

The primary outcome was the 42-day mortality. The secondary outcome was failure to control bleeding, defined by death within 5 days, a 3 g/dL drop in hemoglobin within 24 h after initial hemostasis with endoscopic therapy, and/or failure of endoscopic hemostasis within the first 24 h. Exposure was MTP. The primary indication for massive transfusion was any situation that resulted in acute blood loss and hemodynamic instability. In our institution, when MTP is activated, the blood bank is contacted via a phone or an electronic medical alert system. The MTP calls for a pRBC-to-plasma-to-platelet ratio of 1:1:1. Six units of pRBC, six units of plasma, and six units of platelets, or one unit of plateletpheresis, were released rapidly without cross-matching.

We also collected the following variables by reviewing electronic medical records: age at AVB, gender, cause of liver cirrhosis, initial systolic blood pressure (SBP), initial pulse rate (PR), white blood cell count, hemoglobin count, platelet count, albumin, total bilirubin, aspartate transaminase, alanine transaminase, and model for end stage liver disease (MELD) score. The amount of blood product transfusion was defined as the actual amount of blood transfused within the first 24 h of visiting the emergency room due to AVB in both patients who received and did not receive MTP. The degree of portal vein pressure and advanced cirrhosis in patients was assessed based on the aspartate transaminase to-platelet ratio index (APRI) and the fibrosis 4 (FIB4) index [19,20,21,22].

### 2.3. Statistical Analysis

Values are presented in the form of median (interquartile range) or frequency (percentage). The baseline characteristics of patients who did or did not receive MTP were compared using the *t*-test, Mann–Whitney U test, χ^2^ test, or Fisher’s exact test, as appropriate. Survival was estimated using the Kaplan–Meier method, and the log-rank test was compared between patients who did and did not receive MTP. Prognostic factors were identified using Cox proportional hazards regression analysis performed for the entire study cohort. Multivariable analyses were performed for the whole study cohort using univariable analyses with *p* values < 0.05. VIF 5.0 was used to evaluate multicollinearity. Subgroup analysis was performed according to the initial hemoglobin count, SBP, and MELD score. Additionally, the propensity score was computed for each patient based on age, gender, initial SBP, MELD score, and albumin level. Propensity matching was performed in a 5:1 ratio using the nearest-neighbor distance without replacement of the propensity score (caliper: 0.25) to select two groups of patients with balanced characteristics. Standardized mean differences were used to diagnose the baseline balance. All values were ≤0.1 after matching. Baseline characteristic analysis of the propensity score-matched cohort was performed considering weights and matched pairs that occurred according to the variable matching ratio [23,24]. All statistical analyses were performed using R version 4.0.3 (Vienna, Austria), and a two-tailed *p* < 0.05 was considered significant.

## 3. Results

### 3.1. Overall Cohort Analysis

Nineteen patients underwent MTP during the study. A comparison of the baseline characteristics between patients who did and did not receive MTP is summarized in Table 1. Patients who received MTP had a lower initial SBP (median: 88 vs. 117 mmHg, *p* < 0.001) and higher initial PR (median: 107/min vs. 92/min, *p* = 0.027). Patients who received MTP had lower initial hemoglobin count (mean: 6.96 g/dL vs. 9.06 g/dL, *p* = 0.001) but higher MELD scores (median: 18 vs. 12, *p* < 0.001). Meanwhile, there was no difference between the two groups in the ARPI or FIB-4 index. The actual amounts of transfused blood products were higher in the MTP group: red blood cells (median 7 vs. 0, *p* < 0.001), platelet (median: 6 vs. 0, *p* < 0.001), and fresh frozen plasma (median: 6 vs. 0, *p* < 0.001).

**Figure 2:**
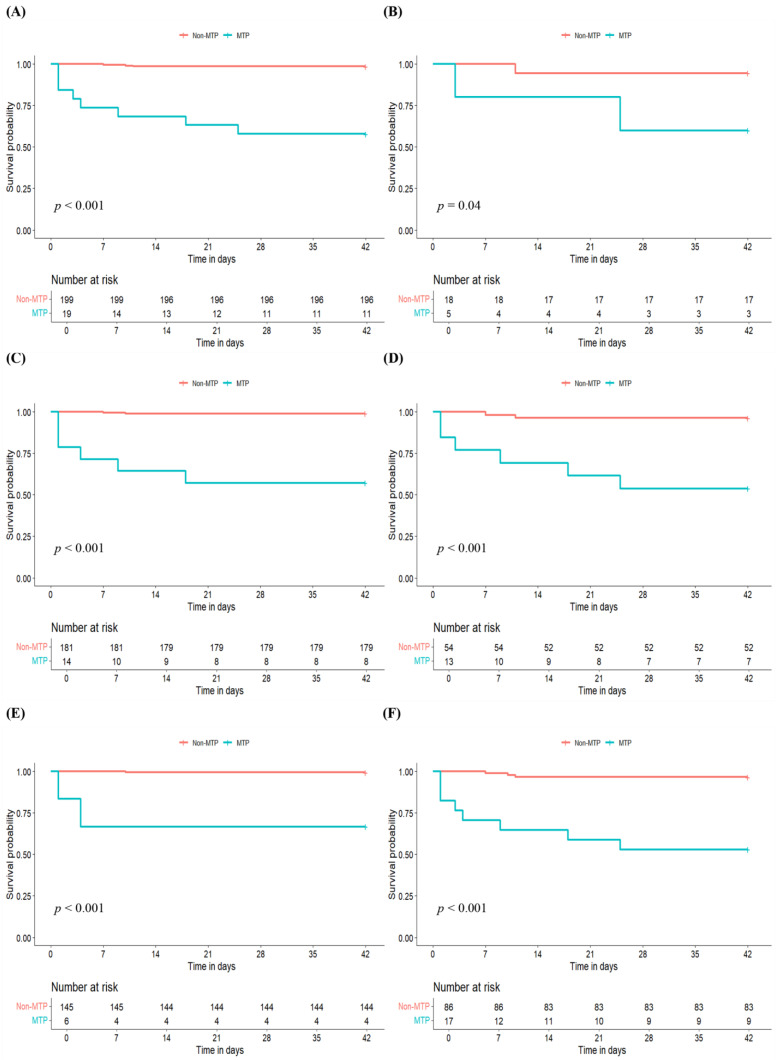
Kaplan-Meier curves of cumulative probability of 42-day mortality in patients receiving MTP compared to those who did not receive MTP. (**A**) Whole cohort, (**B**) Hb < 6.0 g/dL in the whole cohort, (**C**) Hb ≥ 6.0 g/dL in the whole cohort, (**D**) SBP < 100 mmHg in the whole cohort, (**E**) SBP ≥ 100 mmHg in the whole cohort, (**F**) MELD ≥ 13 in the whole cohort. *MTP = massive transfusion protocol; Hb = hemoglobin; SBP = systolic blood pressure; MELD = model for end-stage liver disease*.

The MTP group showed a higher 42-day mortality than the no-MTP group (42.1% vs. 1.5%, *p* < 0.001) (Table 2, Figure 2A). The failure to control bleeding rate was also higher in the MTP group than in the no-MTP group (36.8% vs. 0.5%, *p* < 0.001, Table 2). MTP (HR: 35.47; 95% CI: 9.38–134.14, *p* < 0.001), initial SBP (HR: 0.97; 95% CI: 0.94–0.99, *p* = 0.006), albumin level (HR: 0.22; 95% CI: 0.1–0.5, *p* < 0.001), MELD score (HR: 1.13; 95% CI: 1.06–1.2, *p* < 0.001), and actual amount of transfused blood components (red blood cells [HR: 1.14; 95% CI: 1.09–1.19, *p* < 0.001], platelets [HR: 1.14; 95% CI: 1.08–1.20, *p* < 0.001], and fresh frozen plasma [HR: 1.19; 95% CI: 1.13–1.26, *p* < 0.001]) were associated with 42-day mortality in the univariable analysis (Table 3). Multivariable models were created based on the variables identified as significant in the univariable analysis. As the actual amount of red blood cells transfused and fresh frozen plasma transfused showed multicollinearity, two multivariable models were computed, with one model including red blood cells and platelets and the other model including platelets and fresh frozen plasma (Table 3). In both models, MTP was the only factor associated with 42-day mortality. (HR: 21.05; 95% CI: 3.07–144.21, *p* = 0.002 and HR: 24.01; 95% CI: 3.41–169.31, *p* = 0.001).

### 3.2. Subgroup Analysis

When patients were grouped based on their initial hemoglobin levels (g/dL), the MTP group demonstrated a higher 42-day mortality for those with initial hemoglobin levels < 6 g/dL (40.0% vs. 5.6%, log-rank *p* = 0.04, Figure 2B) and those with initial hemoglobin levels ≥ 6 g/dL (42.9% vs. 1.1%, log-rank *p* < 0.001, Figure 2C). When patients were stratified according to initial SBP, the MTP group showed higher 42-day mortality for patients with initial SBP < 100 mmHg (46.2% vs. 3.7%, log-rank *p* < 0.001, Figure 2D) and for patients with initial SBP ≥ 100 mmHg (33.3% vs. 0.7%, log-rank *p* < 0.001, Figure 2E). When stratified according to MELD score, the 42-day mortality was higher in the MTP group for patients with MELD score ≥ 13 (47.1% vs. 3.5%, log-rank *p* < 0.001, Figure 2F). Among patients with a MELD score < 13, only two patients received MTP. The failure to control the bleeding rate was also higher in the MTP group than in the non-MTP group in the subgroups analyzed (Table 2).

### 3.3. Propensity Score Matched Cohort Analysis

**Figure 3:**
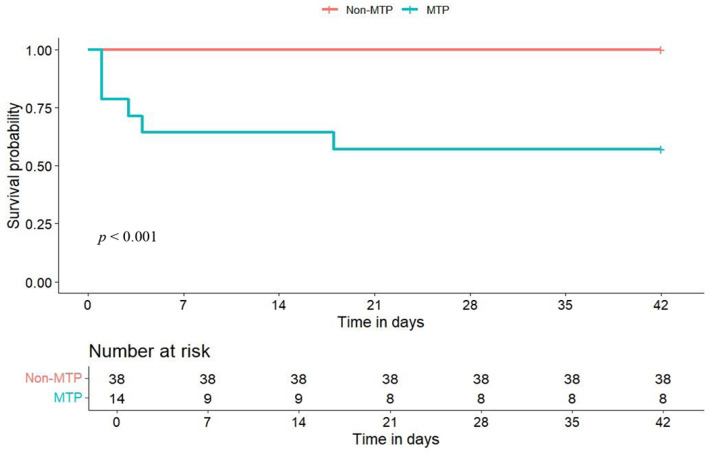
Kaplan-Meier curves of cumulative probability of 42-day mortality in patients receiving MTP compared to those who did not receive MTP in the matched cohort. *MTP, massive transfusion protocol*.

After propensity score matching, 14 patients who received MTP were matched with 38 patients who did not receive MTP (Figure 1). After matching, the majority of the baseline characteristics were balanced, except for the exposure components (Table 1). The actual amount of transfused blood products was higher in the MTP group, including red blood cells (median: 7 vs. 3, *p* = 0.002), platelet (median: 6 vs. 0, *p* = 0.020), and fresh frozen plasma (median: 6 vs. 0, *p* = 0.015). In the propensity score-matched cohort, the 42-day mortality rate (42.9% vs. 0.0%, *p* = 0.001; Table 2, Figure 3) and failure to control bleeding (42.9% vs. 0.0%, *p* = 0.001; Table 2) were higher in the MTP group.

## 4. Discussion

In this study, we found that the 42-day mortality risk increased in AVB patients who received MTP transfusion. The risk of failure to control variceal bleeding (death within 5 days, a 3 g/dL drop in hemoglobin within 24 h, and/or failure of endoscopic hemostasis within the first 24 h) was also higher in AVB patients who received transfusion by MTP. Those who received blood transfusion by MTP were composed of more severely affected patients with hemodynamic instability (lower SBP, higher PR, lower hemoglobin) and those who showed higher MELD scores. Of note, low SBP, low hemoglobin level, and/or MELD score are major predictors of outcomes in AVB [25,26,27]. Hence, we performed a subgroup analysis stratified by SBP, hemoglobin level, and MELD score. In the subgroup analyses, the findings were the same, showing that the MTP group had worse outcomes in all subgroups analyzed. The MTP group also showed an increased risk of 42-day mortality and failure to control variceal bleeding in the propensity score-matched cohort.

To the best of our knowledge, no study has reported the effect of MTP in patients with AVB. However, several studies might explain why MTP was associated with poorer outcomes in the present study. In patients with cirrhosis, blood product transfusion can increase portal pressure [28,29]. Prior studies have shown every 100 mL of blood product transfusion can increase portal pressure by 1.4 ± 0.7 mm of Hg [28,29]. Cirrhotic patients with volume overload have a reduced ability to accommodate fluids after acute volume expansion. These patients might have persistent portal pressure elevation, which can worsen and prolong AVB [9,28]. In addition, several studies confirmed when portal hypertensive-related bleeding occurs, limited transfusion can improve mortality and morbidity [9,30]. In a large randomized clinical trial, patients who received limited blood transfusion (transfusion to maintain 7–9 g/dL when initial hemoglobin was less than 7 g/dL) exhibited a lower mortality rate compared to those who underwent liberal blood transfusion (transfusion when hemoglobin fell below 9 g/dL) (5% vs. 9%, *p* = 0.02). Specifically, among patients with cirrhosis, the incidence of subsequent bleeding was considerably reduced (10% vs. 18%, *p* = 0.01) in the restrictive approach group [9], according to a meta-analysis based on the randomized clinical trial. Restrictive transfusion was linked with decreased risk of all-cause mortality (RR 0.65, 95% CI 0.44–0.97, *p* = 0·03) and overall rebleeding (0.58, 0.40–0.84, *p* = 0.004) when acute upper gastrointestinal bleeding occurred [30]. In previous clinical studies, fresh frozen plasma, platelet, and pRBC transfusions were associated with poor clinical outcomes in patients with AVB [31,32,33]. Notably, compared to non-variceal bleeders receiving a blood transfusion, variceal bleeders had nearly four-fold higher odds of inpatient mortality [33]. In the present study, the amount of blood products transfused was significantly higher in the MTP group (Table 2). The amount of blood product transfused was also a factor associated with mortality within 42 days in the univariable analysis (Table 3). It is plausible that MTP can result in more blood product transfusions, which might aggravate portal hypertension, resulting in increased risks of hemostasis failure, re-bleeding, and mortality rate. Our findings suggest that MTP should not be applied to patients with AVB, even if they show hemodynamic instability until the benefit of MTP is demonstrated by well-designed clinical trials.

This study had some limitations. As this study was not a randomized controlled trial, careful interpretation is needed. Indication bias (indication for MTP activation) was a significant concern in this study. The indication for MTP activation is acute blood loss with hemodynamic instability. The decision to activate the MTP was at the discretion of the attending physician. During the long study period, many doctors managed patients with AVB. The indication of ‘hemodynamic instability’ is a subjective judgment, which can differ by a doctor. As doctors seeing patients with AVB were unaware of this study, this potential bias was independent and nondifferential. We also performed subgroup and propensity score matching analyses to balance the characteristics of patients with or without MTP activation, but the risk of indication bias cannot be completely eliminated in this retrospective study design. Hence, our findings may need to be validated by other studies preferentially through randomized controlled trials. However, randomized controlled trials of AVB with hemodynamic instability require huge medical resources and may be unrealistic in clinical practice. Hence, our analysis might provide some insights into our daily clinical practice despite several inherent limitations. In addition, this study will be valuable as early evidence that can lead to future randomized controlled trials. Next, the amount of crystalloid fluid, which also has volume effects, may influence the treatment outcome of variceal bleeding. Although we sought to investigate the fluid amount given within the initial 24 h, more than half of the patients did not record it because our study focused on patients who visited the emergency room. Therefore, the fluid dose was not included as an evaluation variable. This is also one of the limitations of retrospective research, and if a randomized controlled trial is undertaken in the future, the amount of crystalloid fluid given should also be thoroughly recorded. In addition, the sample size of this study was relatively small. Only 19 patients with AVB received transfusion by MTP during the study period, with a small number of events (11 patients with primary outcome). This requires attention to the interpretation of the data and resulted in a wide range of confidence intervals in the survival analysis (Table 3). Although we collected important covariables that might be associated with the outcomes of this study, the residual confounding variables may exist. However, considering the established pathophysiology that excessive blood transfusions worsen portal hypertension in cirrhotic patients [28,29] and that our data are consistent with prior randomized clinical trials and meta-analysis that identified the benefits of limited transfusions in AVB patients [9,30], the adverse impact of MTP on prognosis may not be merely coincidental. Additionally, given that just 19 individuals were hospitalized in the emergency room and received EVL or EVO throughout an 8-year period, we cannot expect a high number of samples if we wait longer in a single-center study. Based on the findings of this study, which show that applying MTP in varix patients requires caution, we believe that informing them sooner rather than waiting a long time to collect further samples can help improve the prognosis of varix patients. Future multicenter studies with a large number of samples are needed. To the best of our knowledge, this study is the first to evaluate the impact of MTP on AVB, and it will serve as the groundwork for future multicenter studies, large-scale randomized clinical trials, or meta-analyses. Lastly, this study was performed on Korean patients at a single academic institution. Therefore, an external validation is required.

In summary, we did not observe any potential benefits of the MTP. These data call for attention when implementing MTP in patients with AVB. Given the observational nature of this study, further studies are necessary to determine whether MTP can be a treatment option for patients with massive AVB.

## Figures and Tables

**Table 1:** Baseline characteristics of study subjects.

	Whole Cohort			Propensity Score Matched Cohort		
	MTP (*n* = 19)	Non-MTP (*n* = 199)	*p*-Value	MTP (*n* = 14)	Non-MTP (*n* = 38)	*p*-Value
**Male gender, *n* (%)**	12 (63.2)	127 (63.8)	0.95	9 (64.3)	22 (58.9)	0.71
**Age (years)**	53.4 ± 12.6	56.3 ± 12.7	0.35	52.6 ± 11.4	52.4 ± 9.6	0.95
**Etiology, *n* (%)**			0.79			0.84
Alcohol	8 (42.1)	68 (34.2)		6 (42.9)	20 (51.4)	
Viral hepatitis (Hepatitis B virus, Hepatitis C virus)	6 (31.6)	70 (35.2)		4 (28.6)	11 (28.2)	
Others	5 (26.3)	61 (30.7)		4 (28.6)	8 (20.4)	
**Clinical parameters at presentation**						
Initial SBP (mmHg)	88 (62–101)	117 (98–129)	<0.001	88 (67–99)	91 (83–103)	0.48
Initial PR (/min)	107 (88–121)	92 (79–107)	0.027	104(83–109)	95 (81–110)	0.33
**Laboratory values**						
White blood cell count (×10^3^/uL)	9.98 (7.13–11.40)	6.53 (4.41–9.54)	0.012	10.10 (6.18–11.96)	8.55 (6.18–10.97)	0.66
Hemoglobin count (g/dL)	6.96 ± 2.3	9.06 ± 2.49	0.001	7.34 ± 2.35	7.38 ± 2.20	0.95
Platelet count (×10^3^/uL)	83 (51–136)	92 (63–127)	0.81	83 (47–145)	99 (69–115)	0.72
Albumin (g/dL)	2.4 (1.8–3.1)	3.4 (2.9–3.8)	<0.001	2.4 (2.3–3.0)	2.6 (2.3–3.0)	0.95
Total bilirubin (mg/dL)	1.5 (0.8–3.0)	1.6 (0.9–2.7)	0.94	1.5 (0.7–3.0)	2.6 (1.4–4.7)	0.27
Aspartate transaminase (U/L)	48 (27–83)	37 (27–65)	0.48	38 (27–65)	59 (34–82)	0.27
Alanine transaminase (U/L)	21 (14–29)	26 (18–37)	0.14	18 (13–28)	23 (18–31)	0.43
MELD score	18 (14–22)	12 (9–16)	<0.001	16 (13–21)	17 (13–23)	0.77
APRI			0.99			0.24
≤1.0	8 (42.1)	84 (42.2)		7 (50.0)	11 (30.4)	
>1.0	11 (57.9)	115 (57.8)		7 (50.0)	27 (69.6)	
FIB-4			0.99			0.21
≤3.25	7 (36.8)	68 (34.2)		5 (35.7)	7 (17.5)	
>3.25	12 (63.2)	131 (65.8)		9 (64.3)	31 (82.5)	
**Blood product transfusion**						
Red blood cell (Unit)	7 (3–11)	0 (0–2)	<0.001	7 (3–11)	3 (1–3)	0.002
Platelet (Unit)	6 (0–12)	0 (0–0)	<0.001	6 (0–12)	0 (0–5)	0.020
Fresh frozen plasma (Unit)	6 (2–12)	0 (0–0)	<0.001	6 (1–12)	0 (0–3)	0.015

Values are presented in the form of median (interquartile range) or frequency (percentage). *MTP = massive transfusion protocol; SBP = systolic blood pressure; PR = pulse rate; MELD = model for end-stage liver disease; APRI = aspartate transaminase to-platelet ratio index; FIB-4 = fibrosis 4*.

**Table 2:** Death within 42 days and failure to control bleeding according to massive transfusion protocol activation.

	Death within 42-Days			Failure to Control Bleeding		
	MTP *n* = 19 (8.7%)	Non-MTP *n* = 199 (91.3%)	*p*-Value	MTP *n* = 19 (8.7%)	Non-MTP *n* = 199 (91.3%)	*p*-Value
**Overall cohort (*n* = 218)**	8 (42.1%)	3 (1.5%)	<0.001	7 (36.8%)	1 (0.5%)	<0.001
**Subgroup analysis**						
Hemoglobin <6 g/dL (*n* = 23)	2 (40.0%)	1 (5.6%)	0.11	1 (20.0%)	0 (0.0%)	0.22
Hemoglobin ≥6 g/dL (*n* = 195)	6 (42.9%)	2 (1.1%)	<0.001	6 (42.9%)	1 (0.6%)	<0.001
SBP < 100 mmHg (*n* = 67)	6 (46.2%)	2 (3.7%)	<0.001	5 (38.5%)	0 (0.0%)	<0.001
SBP ≥ 100 mmHg (*n* = 151)	2 (33.3%)	1 (0.7%)	0.004	2 (33.3%)	1 (0.7%)	0.004
MELD ≥ 13 (*n* = 103)	8 (47.1%)	3 (3.5%)	<0.001	7 (41.2%)	1 (1.2%)	<0.001
MELD < 13 (*n* = 115)	0 (0.0%)	0 (0.0%)	N/A	0 (0.0%)	0 (0.0%)	N/A
**Propensity matched cohort (*n* = 52)**	6 (42.9%)	0 (0.0%)	0.001	6 (42.9%)	0 (0.0%)	0.001

Values are presented in the form of frequency (percentage). *MTP = massive transfusion protocol; SBP = systolic blood pressure; MELD = model for end-stage liver disease; N/A = not applicable*.

**Table 3:** Factors associated with death within 42 days.

	Univariable Cox Regression		Multivariable Cox Regression Model 1		Multivariable Cox Regression Model 2	
	HR (95% CI)	*p*-Value	HR (95% CI)	*p*-Value	HR (95% CI)	*p*-Value
**Gender**						
Male	Reference					
Female	0.39 (0.08, 1.79)	0.23				
**Age (years)**	1.01 (0.97, 1.06)	0.56				
**MTP**	35.47 (9.38, 134.14)	<0.001	21.05 (3.07, 144.21)	0.002	24.01 (3.41, 169.31)	0.001
**Etiology**		0.28				
Alcohol	Reference					
Viral hepatitis (Hepatitis B virus, Hepatitis C virus)	0.65 (0.18, 2.3)	0.50				
Others	0.19 (0.02, 1.55)	0.12				
**Clinical parameters at presentation**						
Initial SBP (mmHg)	0.97 (0.94, 0.99)	0.006	1.01 (0.99, 1.04)	0.26	1.01 (0.99, 1.04)	0.34
Initial PR (/min)	1.01 (0.98, 1.04)	0.41				
**Laboratory values**						
Hemoglobin count (g/dL)	0.88 (0.69, 1.12)	0.29				
Albumin (g/dL)	0.22 (0.1, 0.5)	<0.001	1.10 (0.37, 3.32)	0.86	0.99 (0.34, 2.87)	0.98
Total bilirubin (mg/dL)	1.1 (0.99, 1.21)	0.07				
**MELD score**	1.13 (1.06, 1.2)	<0.001	1.07 (0.98, 1.17)	0.16	1.07 (0.97, 1.18)	0.18
**Blood product transfusion**						
Red blood cell (Unit)	1.14 (1.09, 1.19)	<0.001	1.05 (0.93, 1.19)	0.44		
Platelet (Unit)	1.14 (1.08, 1.20)	<0.001	1.02 (0.90, 1.15)	0.79	1.06 (0.96, 1.18)	0.26
Fresh frozen plasma (Unit)	1.19 (1.13, 1.26)	<0.001			0.98 (0.84, 1.15)	0.84

*MTP = massive transfusion protocol; SBP = systolic blood pressure; PR = pulse rate; MELD = model for end-stage liver disease*.

## Data Availability

The data underlying this article cannot be shared publicly, given the privacy of the individuals who participated in the study. The data will be shared upon reasonable request by the corresponding author.

## Abbreviations

MTP, massive transfusion protocol; AVB, acute variceal bleeding; RBC, red blood cell; pRBC, packed red blood cells; EVL, endoscopic variceal ligation; EVO, endoscopic variceal obliteration; SBP, systolic blood pressure; PR, pulse rate; MELD, Model for end-stage liver disease; APRI, aspartate transaminase to-platelet ratio index; FIB4, fibrosis 4; Hb, hemoglobin.

## References

[1] Graham D.Y., Smith J.L. (1981). The course of patients after variceal hemorrhage. Gastroenterology.

[2] de Franchis R. (2010). Revising consensus in portal hypertension: Report of the Baveno V consensus workshop on methodology of diagnosis and therapy in portal hypertension. J. Hepatol..

[3] Garcia-Tsao G., Abraldes J.G., Berzigotti A., Bosch J. (2017). Portal hypertensive bleeding in cirrhosis: Risk stratification, diagnosis, and management: 2016 practice guidance by the American Association for the study of liver diseases. Hepatology.

[4] Kim Y.D., Cheon G.J., Kim M.Y., Suk K.T., Baik S.K., Kim D.J. (2012). Changes in the clinical outcomes of variceal bleeding in cirrhotic patients: A 10-year experience in gangwon province, South Korea. Gut Liver.

[5] Seo Y.S., Kim Y.H., Ahn S.H., Yu S.K., Baik S.K., Choi S.K., Heo J., Hahn T., Yoo T.W., Cho S.H. (2008). Clinical features and treatment outcomes of upper gastrointestinal bleeding in patients with cirrhosis. J. Korean Med. Sci..

[6] Hou M.C., Lin H.C., Liu T.T., Kuo BI T., Lee F.Y., Chang F.Y., Lee S.D. (2004). Antibiotic prophylaxis after endoscopic therapy prevents rebleeding in acute variceal hemorrhage: A randomized trial. Hepatology.

[7] Wells M., Chande N., Adams P., Beaton M., Levstik M., Boyce E., Mrkobrada M. (2012). Meta-analysis: Vasoactive medications for the management of acute variceal bleeds. Aliment. Pharmacol. Ther..

[8] Castañeda B., Morales J., Lionetti R., Moitinho E., Andreu V., Pérez-del-Pulgar S., Pizcueta P., Rodés J., Bosch J. (2001). Effects of blood volume restitution following a portal hypertensive-related bleeding in anesthetized cirrhotic rats. Hepatology.

[9] Colomo A., Santaló M., Hernandez-Gea V., Alvarez-Urturi C., Poca M., Graupera I., Gordillo J., Concepción M., Muñiz E., Guarner C. (2013). Transfusion strategies for acute upper gastrointestinal bleeding. N. Engl. J. Med..

[10] Malone D.L., Hess J.R., Fingerhut A. (2006). Massive transfusion practices around the globe and a suggestion for a common massive transfusion protocol. J. Trauma..

[11] Savage S.A., Zarzaur B.L., Croce M.A., Fabian T.C. (2013). Redefining massive transfusion when every second counts. J. Trauma. Acute Care Surg..

[12] Dente C.J., Shaz B.H., Nicholas J.M., Harris R.S., Wyrzykowski A.D., Patel S., Shah A., Vercruysse G.A., Feliciano D.V., Ingram W.L. (2009). Improvements in early mortality and coagulopathy are sustained better in patients with blunt trauma after institution of a massive transfusion protocol in a civilian level I trauma center. J. Trauma.

[13] Borgman M.A., Spinella P.C., Perkins J.G., Grathwohl K.W., Repine T., Beekley A.C., Sebesta J., Jenkins D., Wade C.E., Holcomb J.B. (2007). The ratio of blood products transfused affects mortality in patients receiving massive transfusions at a combat support hospital. J. Trauma..

[14] Holcomb J.B., Tilley B.C., Baraniuk S., Fox E.E., Wade C.E., Podbielski J.M., del Junco D.J., Brasel K.J., Bulger E.M., PROPPR Study Group (2015). Transfusion of plasma, platelets, and red blood cells in a 1: 1: 1 vs a 1: 1: 2 ratio and mortality in patients with severe trauma: The PROPPR randomized clinical trial. JAMA.

[15] Farooq N., Galiatsatos P., Aulakh J.K., Higgins C., Martinez A. (2018). Massive transfusion practice in non-trauma related hemorrhagic shock. J. Crit. Care.

[16] McDaniel L.M., Neal M.D., Sperry J.L., Alarcon L.H., Forsythe R.M., Triulzi D., Peitzman A.B., Raval J.S. (2013). Use of a massive transfusion protocol in nontrauma patients: Activate away. J. Am. Coll. Surg..

[17] The Korean Association for the Study of the Liver (KASL) (2020). KASL clinical practice guidelines for liver cirrhosis: Varices, hepatic encephalopathy, and related complications. Clin. Mol. Hepatol..

[18] Kaplan D.E., Ripoll C., Thiele M., Fortune B.E., Simonetto D.A., Garcia-Tsao G., Bosch J. (2024). AASLD Practice Guidance on risk stratification and management of portal hypertension and varices in cirrhosis. Hepatology.

[19] Kirnake V., Arora A., Sharma P., Goyal M., Chawlani R., Toshniwal J., Kumar A. (2018). Non-invasive aspartate aminotransferase to platelet ratio index correlates well with invasive hepatic venous pressure gradient in cirrhosis. Ind. J. Gastroenterol..

[20] Wang L., Feng Y., Ma X., Wang G., Wu H., Xie X., Zhang C., Zhu Q. (2017). Diagnostic efficacy of noninvasive liver fibrosis indexes in predicting portal hypertension in patients with cirrhosis. PLoS ONE.

[21] Lin Z.H., Xin Y.N., Dong Q.J., Wang Q., Jiang X.J., Zhan S.H., Sun Y., Xuan S.Y. (2011). Performance of the aspartate aminotransferase-to-platelet ratio index for the staging of hepatitis C-related fibrosis: An updated meta-analysis. Hepatology.

[22] Sterling R.K., Lissen E., Clumeck N., Sola R., Correa M.C., Montaner J., Sulkowski M.S., Torriani F.J., Dieterich D.T., Nelson M. (2006). Development of a simple noninvasive index to predict significant fibrosis in patients with HIV/HCV coinfection. Hepatology.

[23] Rao J.N.K., Scott A.J. (1984). On Chi-Squared Tests for Multiway Contingency Tables with Cell Proportions Estimated from Survey Data. Ann. Stat..

[24] Lumley T., Scott A.J. (2013). Two-sample rank tests under complex sampling. Biometrika.

[25] Abraldes J.G., Villanueva C., Bañares R., Aracil C., Catalina M.V., García-Pagán J.C., Bosch J. (2008). Hepatic venous pressure gradient and prognosis in patients with acute variceal bleeding treated with pharmacologic and endoscopic therapy. J. Hepatol..

[26] Goldis A., Lupusoru R., Goldis R., Ratiu I. (2017). Prognostic Factors in Liver Cirrhosis Patients with Upper Gastrointestinal Bleeding. Biol. Med..

[27] Lee J.Y., Lee J.H., Kim S.J., Choi D.R., Kim K.H., Kim Y.B., Kim H.Y., Yoo J.Y. (2002). Comparison of predictive factors related to the mortality and rebleeding caused by variceal bleeding: Child-Pugh score, MELD score, and Rockall score. Taehan Kan. Hakhoe Chi.

[28] Boyer J.L., Chatterjee C., Iber F.L., Basu A.K. (1966). Effect of plasma-volume expansion on portal hypertension. N. Engl. J. Med..

[29] Zimmon D.S., Kessler R.E. (1974). The portal pressure-blood volume relationship in cirrhosis. Gut.

[30] Odutayo A., Desborough M.J., Trivella M., Stanley A.J., Dorée C., Collins G.S., Hopewell S., Brunskill S.J., Kahan B.C., Logan R.F.A. (2017). Restrictive versus liberal blood transfusion for gastrointestinal bleeding: A systematic review and meta-analysis of randomised controlled trials. Lancet Gastroenterol. Hepatol..

[31] Biswas S., Vaishnav M., Pathak P., Gunjan D., Mahapatra S.J., Kedia S., Rout G., Thakur B., Nayak B., Kumar R. (2022). Effect of thrombocytopenia and platelet transfusion on outcomes of acute variceal bleeding in patients with chronic liver disease. World J. Hepatol..

[32] Mohanty A., Kapuria D., Canakis A., Lin H., Amat M.J., Paniz G.R., Placone N.T., Thomasson R., Roy H., Chak E. (2021). Fresh frozen plasma transfusion in acute variceal haemorrhage: Results from a multicentre cohort study. Liver Int..

[33] Radadiya D., Devani K., Rockey D.C. (2022). The impact of red blood cell transfusion practices on inpatient mortality in variceal and non-variceal gastrointestinal bleeding patients: A 20-year US nationwide retrospective analysis. Aliment. Pharmacol. Ther..

